# Structure and mechanism of a bacterial host-protein citrullinating virulence factor, *Porphyromonas gingivalis* peptidylarginine deiminase

**DOI:** 10.1038/srep11969

**Published:** 2015-07-01

**Authors:** Theodoros Goulas, Danuta Mizgalska, Irene Garcia-Ferrer, Tomasz Kantyka, Tibisay Guevara, Borys Szmigielski, Aneta Sroka, Claudia Millán, Isabel Usón, Florian Veillard, Barbara Potempa, Piotr Mydel, Maria Solà, Jan Potempa, F. Xavier Gomis-Rüth

**Affiliations:** 1 Proteolysis Lab; Department of Structural Biology (“María de Maeztu” Unit of Excellence); Molecular Biology Institute of Barcelona, CSIC; Barcelona Science Park, Helix Building; c/Baldiri Reixac, 15–21; E-08028 Barcelona Spain; 2 Structural MitoLab; Department of Structural Biology (“María de Maeztu” Unit of Excellence); Molecular Biology Institute of Barcelona, CSIC; Barcelona Science Park, Helix Building; c/Baldiri Reixac, 15–21; E-08028 Barcelona Spain; 3 Crystallographic Methods; Department of Structural Biology (“María de Maeztu” Unit of Excellence); Molecular Biology Institute of Barcelona, CSIC; Barcelona Science Park, Helix Building; c/Baldiri Reixac, 15–21; E-08028 Barcelona Spain; 4 Laboratory of Microbiology; Faculty of Biochemistry, Biophysics and Biotechnology; Jagiellonian University; Gonostajowa 7; PL-30–387 Kraków Poland; 5 Małopolska Center of Biotechnology, Jagiellonian University; Gonostajowa 7; PL-30-387 Kraków Poland; 6 Institució Catalana de Recerca i Estudis Avançats; Passeig Lluís Companys, 23; E-08003 Barcelona Spain; 7 Department of Oral Immunology and Infectious Diseases; University of Louisville School of Dentistry; Louisville, KY 40202 USA; 8 Broegelmann Research Laboratory; Department of Clinical Science; The Gade Institute; University of Bergen; N-5021 Bergen Norway

## Abstract

Citrullination is a post-translational modification of higher organisms that deiminates arginines in proteins and peptides. It occurs in physiological processes but also pathologies such as multiple sclerosis, fibrosis, Alzheimer’s disease and rheumatoid arthritis (RA). The reaction is catalyzed by peptidylarginine deiminases (PADs), which are found in vertebrates but not in lower organisms. RA has been epidemiologically associated with periodontal disease, whose main infective agent is *Porphyromonas gingivalis*. Uniquely among microbes, *P. gingivalis* secretes a PAD, termed PPAD (*Porphyromonas* peptidylarginine deiminase), which is genetically unrelated to eukaryotic PADs. Here, we studied function of PPAD and its substrate-free, substrate-complex, and substrate-mimic-complex structures. It comprises a flat cylindrical catalytic domain with five-fold α/β-propeller architecture and a C-terminal immunoglobulin-like domain. The PPAD active site is a funnel located on one of the cylinder bases. It accommodates arginines from peptide substrates after major rearrangement of a “Michaelis loop” that closes the cleft. The guanidinium and carboxylate groups of substrates are tightly bound, which explains activity of PPAD against arginines at C-termini but not within peptides. Catalysis is based on a cysteine-histidine-asparagine triad, which is shared with human PAD1-PAD4 and other guanidino-group modifying enzymes. We provide a working mechanism hypothesis based on 18 structure-derived point mutants.

L-Citrulline (*N*^5^-carbamoyl-L-ornithine) is a non-proteinogenic amino acid that is an intermediate in the Krebs-Henseleit urea cycle in animals^1^. It is produced by the enterocytes of the small bowel in humans and its accumulation in plasma can cause citrullinemia, an autosomal recessive disorder characterized by increased citrulline secretion in the urine and neuropsychiatric symptoms^2^. Citrulline is also a biological precursor for nitric oxide and its therapeutic administration has been proposed for the mitochondrial MELAS syndrome^3^. Citrulline further results from free arginine by citrullination, which entails replacement of the guanidino group with an ureido group through deimination. This removes the positive charge of the arginine side chain and liberates ammonia.

Of greater physiological relevance, however, is the citrullination of arginines in peptides and proteins through post-translational modification^4^,^5^. Given the limited number of genes in the genomes of higher organisms, such post-translational modifications increase the structural and functional diversity of the proteomes^4^. Citrullination may result in changes in fold, function and half-life of proteins and peptides, and the reaction is catalyzed in a calcium-dependent manner by peptidylarginine deiminases (PADs). These occur only in vertebrates, where five close paralogs (PAD1-PAD4 and PAD6) have been described^6^,^7^,^8^. Their activity is essential for skin keratinization, neuron insulation, and plasticity of the central nervous system as well as histone core-protein regulation^5^,^7^. Furthermore, through involvement of PADs in apoptosis, autophagy, and NETosis, citrullination plays a major role in the immune system.

However, citrullination also has an established role in pathology, which has lately catapulted interest in the reaction since increased levels of citrullinated proteins are found in several if not all inflammatory diseases^9^ and have been directly implicated in Alzheimer’s disease, prion diseases, psoriasis, multiple sclerosis and tumorigenesis^5^. In a specific genetic background, citrullinated proteins act as autoantigens to generate anti-citrullinated protein antibodies, which participate in an abnormal autoimmune response, a hallmark of rheumatoid arthritis (RA^10^). The latter is a common systemic disease affecting ~1% of the general population in the developed world that is characterized by chronic inflammation of the synovial joints, eventually leading to progressive joint destruction and, despite many years of intensive research, its mechanisms of disease progression are still poorly understood. As to etiology, genetic factors, environmental influences—such as smoking and oral contraceptives—, and concomitant microbial infections are risk factors for developing RA^11^.

Inflammation is also a hallmark of chronic periodontal disease (PD), which is among the most prevalent infectious diseases of mankind^12^. In its severe form, the disease affects the gums of 10–15% of adults, potentially leading to tissue destruction and tooth loss^13^. Its major causal agent is *Porphyromonas gingivalis*, a bacterium that is also implicated in cardiovascular diseases, respiratory diseases, diabetes, osteoporosis, and pre-term low birth-weight. More recently, epidemiological studies have further reported an increased prevalence of PD in RA^14^,^15^, which is consistent with the antique claim made by Hippocrates ~2,400 years ago that removal of bad teeth cures arthritis^16^.

Within the virulence-factor armamentarium of *P. gingivalis* are several secreted cysteine peptidases such as lysine (Kgp) and arginine gingipains A and B (RgpA and RgpB). These are cysteine endopeptidases cleaving after lysines and arginines, respectively, and they participate not only in nutrient acquisition but also in host-tissue destruction and defense inactivation^17^. Uniquely among microbes to date, *P. gingivalis* also produces a secreted PAD (called PPAD^18^), which protects *P. gingivalis* during acidic cleansing in the mouth through ammonia generated during host and endogenous protein citrullination^19^. PPAD does not require calcium for catalysis^20^ and is genetically unrelated with animal PADs and, like the latter and cysteine peptidases, its main catalytic residue is a cysteine (C^351^ in PPAD^20^).

Host PADs process arginines within polypeptide chains but not at their termini, i.e. they are efficient endodeiminases but poor exodeiminases^4^. In contrast, PPAD citrullinates C-terminal arginines like those generated by the prior action of Rgps^17^, which may be facilitated by the surface co-localization of Rgps and PPAD^20^. In this way, PPAD complements endogenous PADs and creates new exogenous epitopes for autoimmune response, which have been associated with RA disease progression^15^. Taken together, all these results suggest that the link between RA and *P. gingivalis*-induced PD may result from PPAD-mediated citrullination^15^.

To shed light on the molecular aspects of this key enzyme for pathogenicity, we analyzed the structure and function of PPAD in various functional states and proposed a working model for the enzyme based on mutational studies, which places PPAD in a wider context with PADs and functionally more distant enzymes.

## Results and Discussion

### Molecular structure of PPAD

PPAD was recently reported to belong to a family of secreted *P. gingivalis* proteins, which includes Kgp and Rgps^20^. These proteins possess a ~75-residue C-terminal domain (CTD) for maturation and translocation through the outer membrane *via* the PorSS, PerioGate or Type-IX secretion system, which removes the CTD upon secretion^21^. Accordingly and similarly to Kgp and Rgps, full-length PPAD would span a pro-peptide, a catalytic domain (CD), an immunoglobulin-superfamily domain (IgSF), and a CTD^18^. We obtained a fragment of PPAD by homologous overexpression in *P. gingivalis* that was equivalent to the purified form from *P. gingivalis* supernatant^18^ and, thus, lacked the pro-peptide and the CTD (residues A^44^-A^475^ ; see Table 1). We solved three distinct structures to high resolution from different protein preparations, which crystallized in different space groups: substrate-free (to 1.5 Å resolution), a substrate-mimic (1.4 Å), and a substrate complex (1.8 Å; see Table 2).

**Figure 1:**
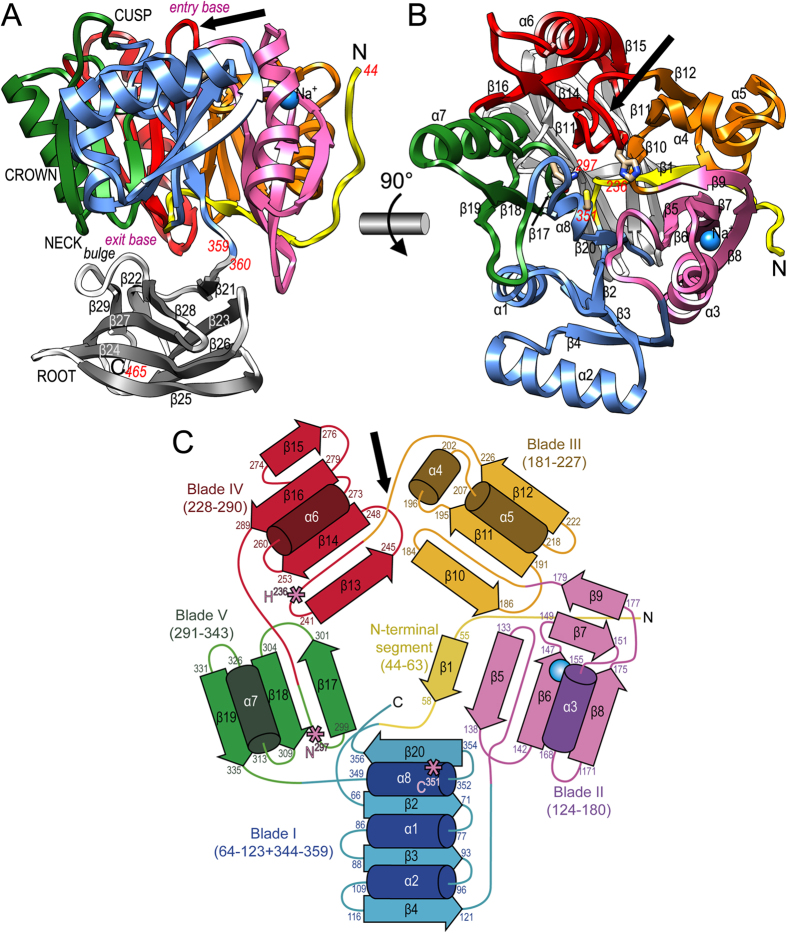
Overall structure and topology of PPAD. (**A**) Ribbon-type plot of PPAD in a lateral view revealing its tooth-like shape, which consists of regions assignable to cusp, crown, neck and root. The upper N-terminal cylindrical catalytic domain (CD; residues 44–359; top entry base and bottom exit base) is shown with the N-terminal segment in yellow and each of its constituting blades (I to V) in one color (blue, magenta, orange, red, and green). The C-terminal IgSF-like domain (residues 360–465) is shown in grey for its β-strands (labeled β22-β29) and white for loops and coils. A sodium ion is shown as a blue sphere and a black arrow pinpoints the Michaelis-loop. (**B**) Top view onto the entry base of the CD cylinder after a horizontal 90°-rotation of (A). The helices (α1-α8) and strands (β1-β20) of the CD are labeled. Catalytic-triad-residue (C^351^, H^236^ and N^297^) side chains are shown and labeled in red to highlight the active site in the center of the α/β-propeller. A black arrow pinpoints the Michaelis-loop. (**C**) Topology scheme of the five-bladed PPAD CD with strands as arrows and helices as cylinders with their respective limiting residues; coloring as in panels (A) and (B). The three catalytic residues of (B) are shown as pink asterisks, and the Michaelis-loop is denoted by a black arrow.

**Figure 2:**
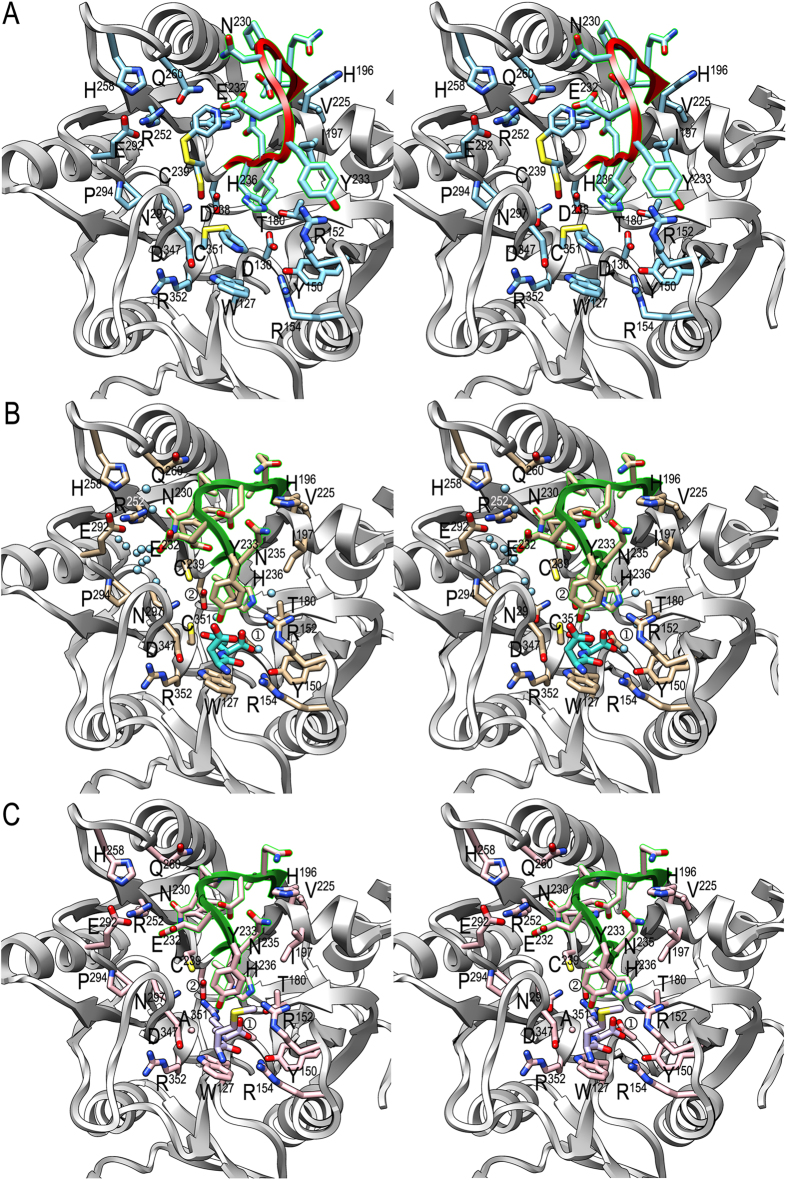
Active-site architecture. (**A**) Stereo image of substrate-free PPAD CD, which actually corresponds to a thiopyridine modified state, with the Michaelis-loop (V^226^-V^237^) shown in red. Selected side chains are displayed with their carbons in light blue and labeled. (**B**) Same view as in (**A**) of the substrate-mimic complex, with the Michaelis-loop in green, unmodified cysteines, side-chain carbons in tan and relevant solvent molecules to illustrate the NH_3_-exit/H_2_O-entry and hydroxide channels as spheres in light blue (see also Fig. 4a). The bound aspartate-glutamine dipeptide is further shown with its carbons in turquoise. ① labels D^130^ and ② labels D^238^. (**C**) Same view as in (A) and (B) of the substrate complex, with the Michaelis-loop in green, unmodified cysteine C^239^ (C^351^ is replaced by alanine), and side-chain carbons in pink. The bound methionine-arginine dipeptide is further depicted with its carbons in purple. ① labels D^130^ and ② labels D^238^.

The PPAD two-domain moiety (CD *plus* IgSF; Fig. 1) shows approximate maximal dimensions of 55 Å(height)×57 Å(width)×50 Å(depth) according to the orientation of Fig. 1a and lacks any bound calcium ion, thus explaining why it is not needed for activity. Overall, it resembles a tooth—with the 316-residue CD featuring the crown and IgSF the root—, which is reminiscent of the gross overall shape of Kgp and RgpB despite completely different functions and CD architectures (see Fig. 2b in^22^ and Fig. 2a in^23^). The neck is the interface between the two domains, and the active site is at the cusp, on the grinding surface (see below). The CD (A^44^-K^359^; see Fig. 1a–c) comprises eight helices and 20 β-strands and is a flat cylinder made up by a distorted five-fold α/β-propeller arranged around a central shaft. The PPAD CD cylinder has an upper entry base, which coincides with the tooth cusp, and an opposite lower exit base at the neck (Fig. 1a). Around the shaft, five propeller blades (I to V) spanning between 47 (blade III) and 76 (blade I) residues are sequentially arranged counterclockwise according to Fig. 1b,c. Each blade starts on the entry base with a loop connected to the previous blade and consists at least of a three-stranded twisted β-sheet with an inner, a middle and an outer strand, *plus* one helix. The inner strand runs across the cylinder to the exit base paralleling the central shaft. A short loop links the inner strand with the antiparallel middle strand, which runs in the opposite direction towards the entry base. This strand is connected through another loop with the helix, which lines the cylinder side wall. Finally, the helix is linked to the outer strand, which parallels the middle strand and likewise lines the cylinder side wall. Into this minimal architecture—found only in blade V (Fig. 1c)—, additional structural elements are inserted in each blade, thus accounting for overall blade asymmetry and chain lengths. In particular, a sodium ion is pinched by the inner strand and the consensus helix of blade II and is bound in an octahedral manner by six oxygens at distances of 2.30–2.63 Å: D^148^O, D^158^O, and two solvent molecules coplanar with the cation; and apically by D^147^Oδ1 and D^158^Oδ1.

Preceding the first blade, an N-terminal extension (A^44^-R^63^) is found attached to blade II on the cylinder side wall running from the entry base to the exit base (Fig. 1a–c). Here, the polypeptide undergoes a kink and, paralleling the inner strand of blade II, runs along the exit base between blades II and III until the central shaft. There, it runs upward as the middle strand of blade I. The C-terminal segment after blade V enters blade I and provides an extra helix followed by the inner strand of the consensus topology, thus internally fastening the molecule like a Velcro strip. Thereafter, the polypeptide reaches the exit base of the CD and enters the C-terminal 106-residue IgSF domain.

The IgSF domain (G^360^-E^465^) is a distorted 4 + 5-stranded β-sandwich (strands β21-β29) with an antiparallel back sheet (β21↓-β23↑-β26↓-β25↑) and a mixed front sheet (β22↓-β28 + β29↓-β27↑-β24↓-β25↑) whose planes are rotated away by ~25°. The right lateral flank of the domain is closed by strand β25, whose N-terminal and C-terminal halves participate, respectively, in the front and back sheets. The left lateral flank is much wider and open, and contains a bulge dividing the second strand from the left of the front sheet in two (β28 and β29). This bulge interacts with the exit base of the CD (Fig. 1a). Overall, the topology and strand-connectivity of PPAD IgSF is strongly reminiscent of that of Kgp and RgpB^22^,^23^,^24^, but while the width (~25 Å) and depth (~20 Å) of the domains are similar, the length—along the strands of the sheets—is much greater in PPAD than in gingipains (~50 Å *vs*. ~35 Å).

### Active site of PPAD

The propeller shaft in PPAD is rather solid, with a shallow cavity on its entry base coinciding with the tooth cusp that contains the active site (Fig. 1a,b). The latter is mainly a narrow funnel-like hole, which accommodates an arginine side chain of a peptidic or protein substrate. It is framed by the main chain and side chains of the loops connecting blades I and II, II and III, III and IV, and V and I; segment β7-loop β7α3-α3 of blade II; and helix α8 of blade I (Figs 1b and 2a–c). In the substrate-free structure, which was obtained with DTDP-treated wild-type (wt) protein, catalytic C^351^, nearby C^239^ and distal C^462^ residues are covalently modified by what was conservatively interpreted as a 4-thiopyridyl moiety. The C^239^ side chain is even found in two alternate conformations, one bound to thiopyridine and the other with the sulfur as sulfoxide (Fig. 2a). This indicates overall flexibility of active site residues in PPAD due to the absence of a bound substrate and suggests that the covalent modifications of the Sγ atoms do not distort the general unbound conformation of PPAD. In addition, segment V^226^-V^237^ of the loop connecting blades III and IV, hereafter the “Michaelis loop”, is in an open conformation, thus consistent with a structure that can bind a substrate. In particular, Y^233^ at the most exposed part of the loop points to bulk solvent (Fig. 2a). We further obtained a substrate-mimic complex of DTDP-untreated wt PPAD with dipeptide aspartate-glutamine, and a true substrate complex of DTDP-untreated PPAD–C^351^A with dipeptide methionine-arginine. The identification of the peptides was based on high-resolution Fourier maps and surrounding binding partners. The complexes were obtained serendipitously, and all attempts to obtain complexes with other substrates or products failed. We hypothesize that DTDP-treatment precludes substrate binding and, thus, protects the unbound conformation, while lack of such treatment causes the enzyme to trap substrates or mimics during biosynthesis or purification. The complex structures are equivalent, including the backbone of the bound dipeptides (Fig. 2b,c), except for some minimal displacement and the differing side chains (Fig. 2b,c), so the substrate complex is taken hereafter as reference except for issues dealing with C^351^Sγ, for which the substrate-mimic complex will be referred to.

The complex structures allowed us to identify PPAD elements required for substrate binding and catalysis. Comparison with the substrate-free structure revealed overall coincidence of the complexes except for the rearrangement of the Michaelis loop (maximal displacement 7.5 Å at N^230^Cα), which adopts a closed conformation that traps the substrate arginine side chain (Fig. 2b). This causes H^196^ to be rotated ~100° around its χ_1_ angle toward bulk solvent and Y^233^ to be displaced by 4.1 Å and slightly reoriented for its side chain to bind the substrate (see below). Michaelis-loop rearrangement further causes ~90° rotation of H^236^ around its χ_2_ angle, so that its Nδ1 atom is apical to the guanidinium plane (3.2 Å away from arginine Cζ atom; see below) and may play a role in catalysis (see below). Catalytic C^351^Sγ, at the bottom of the cleft, occupies the opposite apical position and the atom is further in binding distance from N^297^Oδ1 (3.3 Å), which could potentially assist in catalysis (see below). N^297^Nδ2, in turn, is in binding distance of D^238^Oδ1 (3.3 Å). The guanidinium group is further tightly bound by D^238^ through a double salt bridge with arginine Nη1 and Nη2 atoms (2.9 Å and 3.0 Å), by the main-chain carbonyl of T^346^ (3.2 Å away from atom Nη1), and by D^130^ through a second double salt bridge with arginine Nε and Nη2 atoms (2.8 Å and 2.9 Å). D^130^ becomes rotated around its χ_1_ angle by ~60° upon substrate binding, thereby exchanging its tight hydrogen bond with T^180^Oγ1 (2.6 Å) in the substrate-free structure with binding of the substrate guanidinium group. These five interactions of the guanidinium group occur roughly in the plane of the latter. The aliphatic part of the arginine is bound between the hydrophobic side chains of I^234^ and W^127^ (both 3.7 Å apart). The latter is held in place by a hydrogen bond between its Nε1 atom and D^347^Oδ1 (2.9 Å), which also confers to the tryptophan a potential role in overall structure maintenance due to its stabilizing function of the loop connecting blades V and I (see below). Interestingly, two small solvent-accessible channels are found roughly on either side of the guanidinium plane, on the right and the left in Fig. 2b. The left channel, hereafter “NH_3_-exit/H_2_O-entry channel,” is framed by segments T^290^-N^297^, N^230^-E^232^, G^345^-T^346^, R^252^, H^258^, and, in particular, C^239^, which is closest to the substrate guanidinium and thus acts as a gatekeeper of the channel. The right channel, in turn, is shallower and does not reach the substrate but rather H^236^Nε2, which is bound to two solvent molecules (see below). This “hydroxide-entry channel” is framed by Y^233^-N^235^, N^151^-R^152^, I^197^, and E^201^.

On the outer border of the active-site cavity, the main chain of the substrate is tightly bound through six interactions. The C-terminal carboxylate is linked by a double salt bridge with R^152^Nη2 (3.0 Å) and Nε (2.8 Å). In addition, one of the carboxylate oxygens is further bound by R^154^Nη1 (2.9 Å) and the other by Y^233^Oη (2.8 Å). The latter atom also binds the main-chain amido nitrogen (3.4 Å), and the preceding peptide carbonyl is hydrogen-bonded by R^154^Nη2 (2.7 Å). This interaction seems to be the main factor responsible for the selectivity of PPAD for peptidylarginines over free arginine^18^. In addition, these interactions draw an intricate network to fix the substrate in the cleft, which makes it difficult to imagine how a substrate with C-terminal extension to the arginine, i.e. an endodeiminase substrate, would be bound by PPAD, as a C-terminally extended peptide would collide with Y^233^ and R^152^ side chains (Fig. 2b,c). Finally, lack of specific interactions with atoms upstream of the last peptide bond of the substrate accounts for PPAD’s capacity to non-specifically turn over both peptides and proteins with C-terminal arginines, i.e. as long as the C-terminus is freely accessible.

### Peptidylarginine deiminase activity and mutant studies ***in vitro***

**Figure 3:**
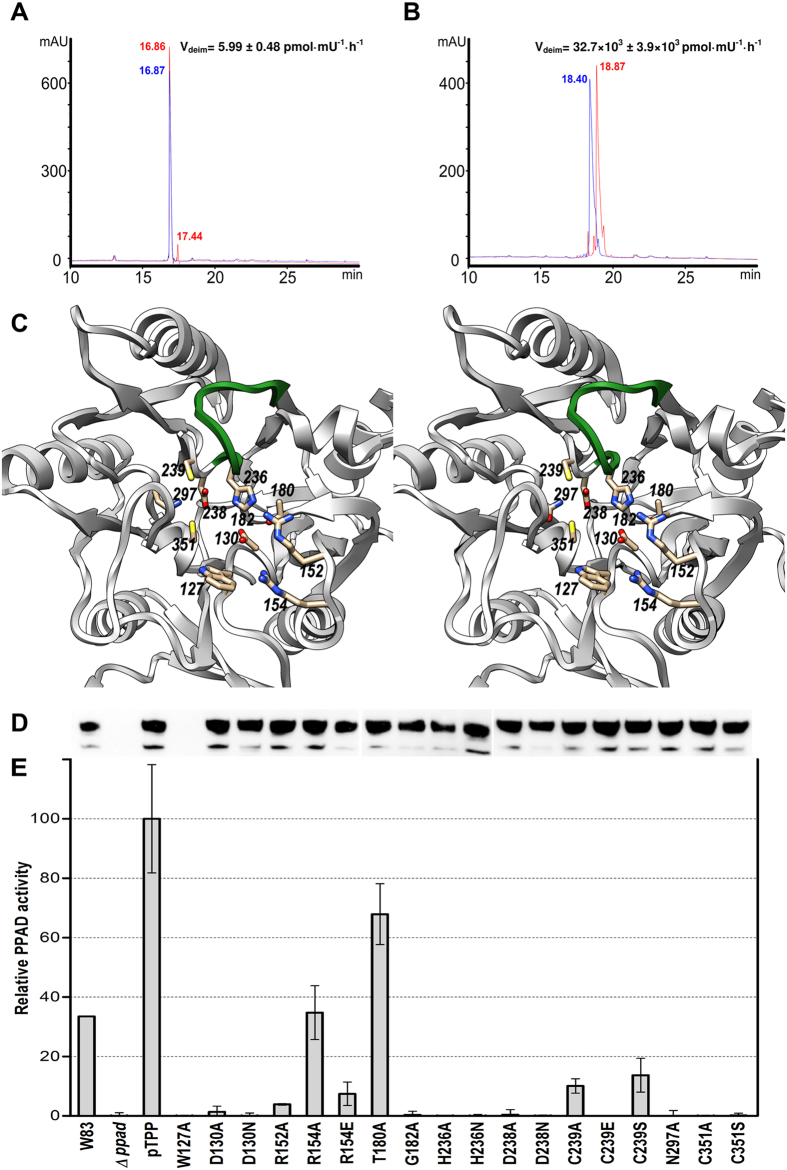
PPAD activity assays. (**A**) Endo- and (**B**) exo-deimininase activity assays *in vitro* of *P. gingivalis* W83 wt PPAD against peptides of sequence G-F-S-P-F-R-S-S and P-P-G-F-S-P-F-R, respectively. Peptides are shown before (blue HPLC chromatograms) and after reaction with PPAD (red HPLC chromatograms). Citrullination caused a shift in the retention time of the peptides when compared with the original ones and was confirmed by mass spectrometry. Based on peak integration, the velocity of reaction was calculated for both peptides, which indicated that peptidylarginine exodeiminase activity of PPAD was nearly 5,500 times higher than endodeiminase activity based on reaction velocity (32,700 *vs.* 6 pmol·mU^−1^·h^−1^). (**C**) Stereo image depicting the 11 positions subjected to point mutagenesis and activity measurements (see (C) and (D)). The Michaelis-loop is shown in green for reference. (**D**) PPAD expression monitoring through Western-blot analysis of whole bacterial cultures resolved on SDS-PAGE and probed with an anti-PPAD antibody. The samples correspond to those of the abscissa of panel (E). (**E**) Relative deiminase activity in front of *N*-acetylarginine of wt W83 strain supernatant (W83), of a PPAD-deletion mutant strain (*Δppad*), of the latter containing plasmid pTPP for wt PPAD overexpression (pTPP; reference 100%), and a cohort of single point mutants around the active site encoded by pTPP variants.

PPAD is an efficient deiminase of peptides including bradykinin and benzoylglycylarginine^18^, EGF and anaphylatoxin C5a^20^, and Rgp-derived fibrinogen peptides, as well as a large set of bacterial cell-envelope proteins truncated by Rgps. To provide additional data on the endo- and exodeiminase activities of PPAD *in vitro*, we tested two octapeptides of equivalent charge derived from the physiologically-relevant bradykinin precursor sequence, respectively with an arginine at position six (G-F-S-P-F-R-S-S; Fig. 3a) and at the C-terminus (P-P-G-F-S-P-F-R; Fig. 3b). We found that peptidylarginine exodeiminase activity of PPAD was nearly 5,500 times higher than endodeiminase activity. This supports the structural findings above. In addition, detailed inspection of the final refined Fourier maps and thermal displacement parameters of atoms Nη1, Cζ, and Nη2 of all twelve internal arginines of the substrate-mimic complex of PPAD, which was refined with data to very high resolution (1.4 Å; see Table 2), revealed no significant evidence for citrullination, strongly suggesting that PPAD produced by homologous overexpression in *P. gingivalis* is not endocitrullinated. Taken together, all these findings strongly support that PPAD is an exodeiminase, as already suggested in the initial report in 1999^18^, and that N-terminal arginines of peptides, endosubstrates and standalone arginines are only modified at a much lower rate, if at all^18^.

In order to discern the functional role of the distinct residues identified in the structures above, we constructed a cohort of 18 single-point mutants of positions 127, 130, 152, 154, 180, 182, 236, 238, 239, 297 and 351 (Table 1 and Fig. 3c) and assessed the deiminase activity of the respective cell cultures relative to the wt. Difficulties in the production of wt and mutant PPADs, which were obtained from *P. gingivalis* cultures, precluded more extensive enzymatic analyses with purified protein. Mutant expression levels were equivalent to those of the wt as monitored by Western-blot analysis, thus pointing to properly folded proteins. The sole exception was W^127^A, which in accordance with a structural role in addition to a substrate-binding role (see above), was not produced in detectable amounts (Fig. 3d). As expected, activity was completely abolished when mutating catalytic C^351^—to either alanine or serine—, but also when replacing D^238^ or H^236^—to either alanine or asparagine—, which participate in substrate guanidinium Cζ atom pinching (Fig. 3e). N^297^, in binding distance of C^351^Sγ, likewise yielded an inactive enzyme when replaced with alanine. D^130^, which strongly binds the guanidinium, is also indispensable, and C^239^, the gatekeeper of one of the two solvent channels, is also relevant as its alanine and serine mutants were just ~8% active and its glutamate mutant was completely inactive. G^182^, in turn, is required to be side-chain depleted as it shuts the bottom of the pocket and is close to H^236^ and D^130^. Its replacement with alanine yielded a complete loss of activity. In contrast, T^180^, which interacts with the two latter residues, is unessential and its alanine mutant still had ~66% activity. Interestingly, R^152^, which establishes a double salt bridge with the substrate carboxylate, is absolutely indispensable for activity, while the second carboxylate-binding arginine, R^154^, is less relevant, with its alanine mutant still showing ~30% activity. Its glutamate mutant, however, which introduces a negative charge next to the also negatively-charged substrate carboxylate, thus causing repulsion, was less than ~10% active.

### Mechanism of peptide citrullination by PPAD

**Figure 4:**
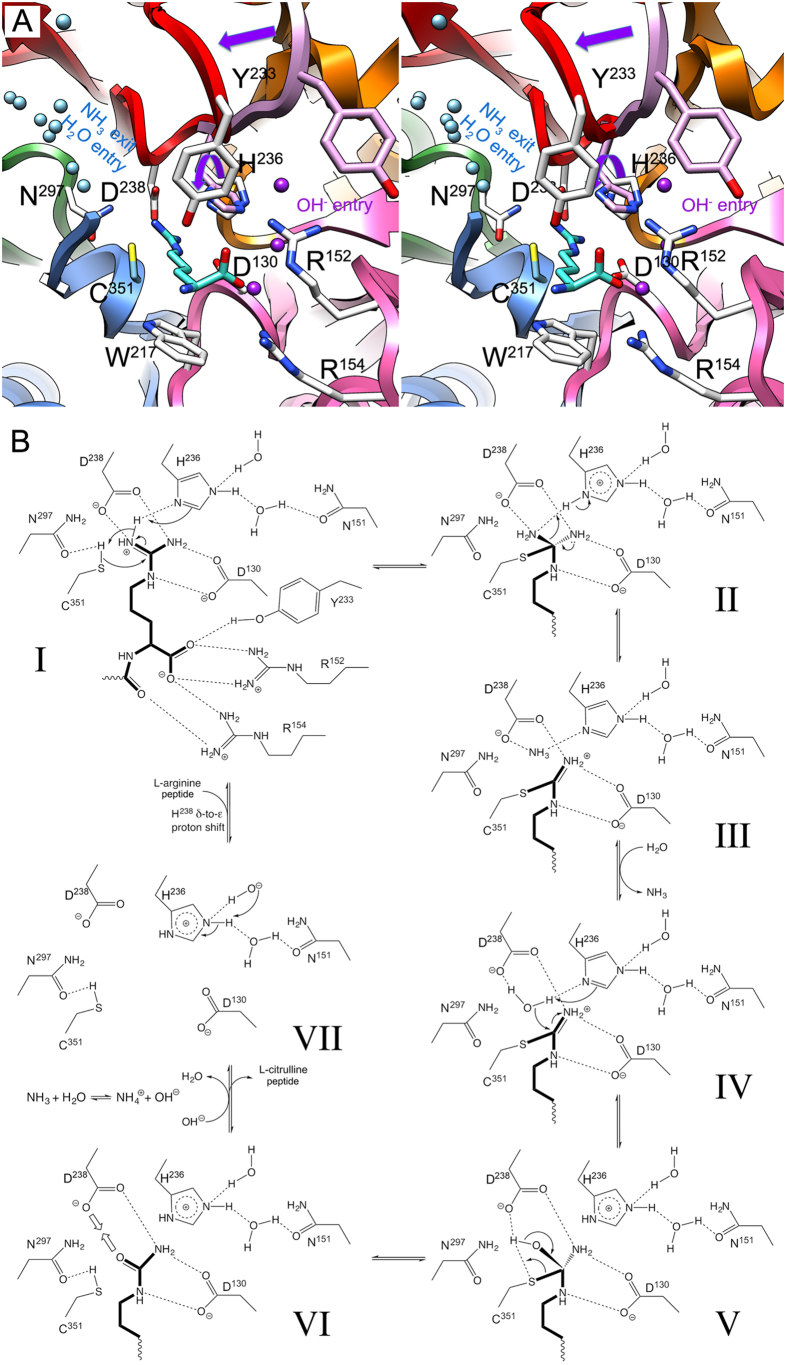
Proposed peptide citrullinating mechanism of PPAD. (**A**) Composite picture in stereo of the active site of PPAD (see also Fig. 2) based on the substrate-mimic complex ribbon plot colored as in Fig. 2b. Only elements engaged in substrate binding and catalysis are depicted. Residue side chains taken from the substrate-mimic complex are shown with carbons in light blue (C^351^), those from the substrate complex in white (Y^233^, H^236^, D^238^, N^297^, R^152^, R^154^, and W^217^), and those from the unbound structure in pink (Y^233^ and H^236^). The Michaelis loop is shown in the open conformation of the unbound structure in pink and in the occluded conformation of the substrate(-mimic) complexes in red, a purple straight arrow highlights the rearrangement upon substrate binding. The substrate arginine depicted belongs to the substrate complex (carbons in turquoise). Solvent molecules from the substrate-mimic complex in light blue highlight the NH_3_-exit/H_2_O-entry channel on the left and those in purple the hydroxide-entry channel on the right. The rotation of the H^236^ side chain from the substrate-unbound to the bound conformation is pinpointed by a curved purple arrow. (**B**) Proposed biochemical mechanism of action of an enzymatic activity cycle in seven steps (I to VII). The substrate arginine and product citrulline are shown with bonds in bold, hydrogen bonds are shown as dashed lines.

We propose the following chemical mechanism of function of PPAD, which includes a catalytic triad (C^351^-H^236^-N^297^) and seven steps proceeding over two tetrahedral and one planar-thiouronium covalent reaction intermediates (Fig. 4a,b).

In the substrate-free state, the Michaelis-loop containing Y^233^ is in an open conformation, which enables peptides with a C-terminal arginine to be accommodated at the active site. The arginine becomes firmly anchored through electrostatic interactions of the guanidinium group with the side chains of D^238^ and D^130^, being positioned in an extended conformation and appropriately oriented for catalysis. In addition, R^152^ and R^154^ bind the C-terminal carboxylate of the arginine and the carbonyl of the preceding peptide bond. Moreover, formation of this Michaelis complex (Fig. 4b, I) entails major rearrangement of the Michaelis loop, which occludes the active site and causes Y^233^ to further bind the C-terminal carboxylate of the substrate. Rearrangement further entails that the side chain of H^236^ is rotated, as a result of which the plane of the guanidinium group becomes pinched between H^236^Nδ1 and C^351^Sγ, and H^236^Nε2 is solvent-bound in the hydroxide-entry channel (Fig. 4a,b). This geometry was determinant for the identification of H^236^ as the general base/acid of the mechanism and of the guanidinium Nη1 atom as the nitrogen atom of the leaving ammonia product. In addition, C^351^Sγ is hydrogen-bonded to N^297^Oδ1, which probably enhances the nucleophilicity of the catalytic sulfur. In the first step of the reaction, C^351^Sγ performs a nucleophilic attack on the sp^2^-like planar Cζ atom of the substrate guanidinium (Fig. 4b, I), giving rise to the first neutral tetrahedral reaction intermediate and yielding an sp^3^-like Cζ atom. Concomitantly, H^236^, which acts first as a general base, abstracts a proton from Nη1, and the latter captures the proton from the catalytic thiol group. The histidine is now in a diprotonated state (Fig. 4b, II). The tetrahedral intermediate collapses to a positively-charged planar thiouronium covalent intermediate and ammonia, which receives a proton from H^236^Nδ1, now acting as a general acid (Fig. 4b, II and III). Ammonia leaves the active site through the NH_3_-exit/H_2_O-entry channel (Fig. 4a) and reaches the surface of the enzyme. In the next step, a solvent molecule—probably a water—occupies the former position of ammonia and becomes polarized by the side chain of D^238^ and H^236^Nδ1. The latter again acts as a base and abstracts a proton from the water molecule, which performs a nucleophilic attack on the central carbon of the thiouronium (Fig. 4b, IV). This yields the second neutral intermediate centered on sp^3^-like tetrahedral Cζ and diprotonated H^236^ (Fig. 4b, V). The intermediate itself collapses to a citrullinated product and the intact catalytic cysteine mercapto group, which becomes hydrogen-bonded to N^297^Oδ1. The repulsion between D^238^ and the carbonyl oxygen of the neutral reaction product may provide the driving force for clearance of the latter from the active-site cleft (Fig. 4b, VI). Finally, a hydroxide resulting from the reaction of ammonia with water may enter the active site through the hydroxide-entry channel and replace one of the two solvent molecules bound to H^236^Nε1. The latter histidine transfers a proton to the hydroxide and a proton shift from Nδ1 to Nε2 restores the functional monoprotonated state of H^236^, thus leaving the active site posed for a new round of reaction (Fig. 4b, VII).

### Structural similarity of PPAD catalytic domain

**Figure 5:**
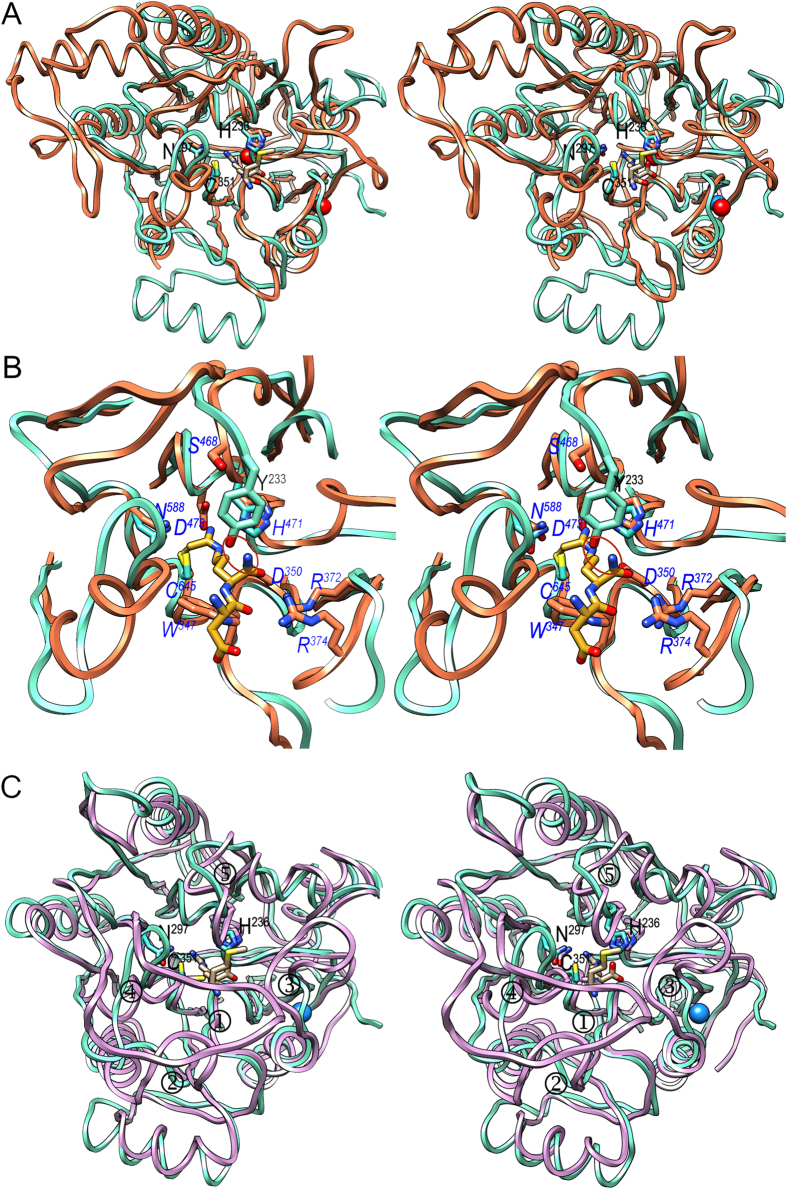
Structural similarities. (**A**) Superposed ribbon-plots in stereo of PPAD in its substrate-mimic complex (cyan) and human PAD4 (coral; PDB >4DKT^54^) as found in its covalent thiouronium reaction intermediate mimic complex. The side chains of the respective catalytic triads (labeled for PPAD only), as well as the two calcium ions of PAD4 (red spheres) and the sodium ion of PPAD (blue sphere) are shown, as is the methionine-arginine dipeptide from the PPAD substrate complex (carbons in tan). Most loops connecting the blades and the consensus secondary elements within each blade differ in length and conformation. (**B**) Close-up of (A). The side chains of the catalytic triad (not labeled) and Y^233^ (labeled in black) of PPAD are depicted (carbons in cyan), as are several representative residues from human PAD4 (carbons in coral; labeled in blue italics) and the covalently bound intermediate (carbons in goldenrod). The mechanistically-relevant equivalent positions (see Fig. 4a,b) in PPAD/human PAD4 (in italics) are C^351^/*C*^*645*^, H^236^/*H*^*471*^, N^297^/*N*^*588*^, D^238^/*D*^*473*^, D^130^/*D*^*350*^, W^217^/*W*^*347*^, Y^233^/*S*^*468*^, R^152^/*R*^*372*^, and R^154^/*R*^*374*^. A red ellipse highlights the clash an endodeiminase substrate would have with PPAD Y^233^. The latter is equivalent to *S*^*468*^ in human PAD4, which allows for free space for C-terminally elongated substrates. (**C**) Same as (**A**) showing PPAD (cyan) and AgDI from *Enterococcus faecalis* (purple; PDB >2JER^26^) as found in a covalent adduct with an agmatine-derived amidine reaction intermediate. The respective catalytic triads are depicted and that of PPAD is also labeled. AgDI main-chain segments diverging from PPAD and mainly accounting for a closed active site are pinpointed (① to ⑤). The mechanistically-relevant equivalent positions (see Fig. 4a,b) in PPAD/AgDI (in italics) are C^351^/*C*^*357*^, H^236^/*H*^*218*,^ N^297^/*N*^*306*^, D^238^/*D*^*220*^, D^130^/*D*^*96*^, and W^217^/*W*^*93*^.

PPAD CD conforms to the structural requirements of the guanidino-group modifying enzyme superfamily (GME; see Fig. 5a–c), which adopts similar five-fold α/β-propeller folds and catalyzes chemical processing of (methylated) guanidine groups as found in the citrullinating GME members: PADs, PPAD, agmatine deiminases (AgDIs), and arginine deiminases (ADIs), which are all dimers or tetramers with the exception of PPAD^25^. AgDIs deiminate isolated agmatine (1-[4-aminobutyl]-guanidine) to *N*-carbamoylputrescine and ammonia as part of mechanisms by which energy is harnessed for growth^26^, and they are missing in higher eukaryotes^25^. ADIs, in turn, citrullinate standalone arginine and protect cells from acidic environments. They are found in plants and microorganisms but are likewise absent from animals^27^. Both families do not have extra domains further to the catalytic α/β-propeller.

To date, only the structures of human PAD2 and PAD4 have been determined among PADs^8^,^28^, and they comprise a ~375-residue calcium-dependent α/β-propeller domain preceded by two IgG domains (see Fig. 1 in^8^ and^28^), which are unrelated to PPAD IgSF further to being all-β protein domains. Among AgDIs, structures have been reported from *Enterococcus faecalis* (*Ef*; Protein Data Bank (PDB) access code 2JER^26^) and *Helicobacter pylori* (*Hp*; PDB 3HVM^29^), and other potential relatives have been deposited with the PDB but not functionally analyzed or published (PDB 2EWO, 1XKN, 1ZBR, 2CMU, 3H7C and 1VKP). Finally, ADI structures have been reported from *Streptococcus pyogenes* (*Sp*; PDB 4BOF^30^), *Pseudomonas aeruginosa* (*Pa*; PDB 1RXX^31^), and *Mycoplasma arginini* (*Ma*; PDB 1S9R^32^). Among all these, closest structural similarity of PPAD is found with AgDIs (Z-score of 35 according to program DALI^33^; see Fig. 5c), followed by PADs (Z = 18–21; Fig. 5a,b) and ADIs (Z = 18–19).

Superposition of the PPAD α/β-propeller on that of human PAD4 (Fig. 5a,b), *Ef*AgDI (Fig. 5c), and *Pa*ADI, *Sp*ADI and *Ma*ADI (data not shown) reveals good overall conservation of the five-blade architectures, although several decorations in the distinct blades of each family account for large differences, especially in the loops surrounding the active-site cleft. In particular, PADs evince a large partially helical insertion between β14 and α6 of PPAD blade IV and lack α2 of blade I (Fig. 5a). ADIs, in turn, evince a large helical sub-domain replacing α2 and β4 of PPAD blade I. In common, all propellers are closed by the blade V-blade I Velcro mechanism (see above and^25^) and the catalytic cysteines and histidines are conserved, as well as the two aspartates anchoring the guanidine group to the bottom of the active site. In addition, PPAD shares with ADIs and PADs the two arginines binding the main chain of the substrate. While these firmly bind the substrate C-terminus in PPAD and ADIs, in PADs they are slightly reoriented and only bind what would be one of the two carboxylate oxygens in addition to the upstream peptide carbonyl (Fig. 5b). This, together with the replacement of PPAD Y^233^ of the Michaelis loop by serine (*S*^*468*^; PAD4; residue numbering of proteins distinct from PPAD in italics) or threonine (*T*^*468*^; PAD2), provides enough space in PADs to allow for a C-terminal extension of the substrate. Furthermore, calcium-dependence of PADs is characterized by several calcium-binding sites^8^,^28^, two of which occur within the propeller domain: one close to the active site with evident implications for function and the other at the domain periphery (Fig. 5a). Interestingly, the latter coincides with the sodium site of PPAD, so a predominantly structural role for both is suggested. In contrast to PADs and PPAD, AgDIs and ADIs, which only process standalone residues, possess completely closed active sites (Fig. 5c).

Most notably, superposition also revealed that all these families possess an equivalent of PPAD asparagine N^297^, i.e. with a potential role in catalysis (PAD2, *N*^*590*^; PAD4, *N*^*588*^; *Ef*AgDI, *N*^*306*^; *Hp*AgDI, *N*^*274*^; *Pa*ADI, *N*^*360*^; *Ma*ADI, *N*^*352*^; and *Sp*ADI, *N*^*355*^). To our knowledge, this was previously unnoticed since this residue, which is strictly conserved across citrullinating GMEs, was merely recognized as an important residue for proper active-site conformation conserved in the consensus helix of blade V of all families (see Fig. 4 in^25^). In PADs, this asparagine is also conserved in distant orthologs from zebrafish and chicken within a shared motif (M/L-V-N-M^34^), which complements the consensus motif encompassing the catalytic cysteine residue (G-E-I/V-H-C-G-T/S). The only notable exception is human PAD6, which lacks both motifs and the calcium sites that are essential for activity in the other paralogs and orthologs^34^. This absence, together with the lack of direct evidence for activity *in vitro* with the assays routinely employed for the other PADs, poses the question as to whether PAD6 is an active peptidylarginine deiminase or whether it may require further factors or interacting partners for activity^35^. In any case, it is likely to follow a different catalytic mechanism.

In all the above structures, the asparagine is at suitable distances and in appropriate orientations to polarize the catalytic cysteine, as found in papain-like cysteine peptidases—in particular, Kgp and RgpB have *N*^*510*^—so we suggest that citrullinating GMEs all have a cysteine-histidine-asparagine catalytic triad as shown for PPAD (see above). However, in contrast to cysteine peptidases, the three residues do not establish a charge-relay system for proton transfer, but rather cysteine-asparagine and histidine act separately on opposite faces of the plane of the guanidinium (Fig. 4a,b).

### Concluding remarks

Structural considerations identified PPAD as a closer relative of AgDIs, which are found across bacteria, than of PADs, which are found only in vertebrates. This, in turn, enables us to hypothesize that PPAD was acquired through horizontal gene transfer of a bacterial single-domain agmatine-citrullinating enzyme. The latter would then have evolved in a different bacterial environment under fusion to two new C-terminal domains like those found in cognate RgpB, to be secreted through a distinct system. This evolution further yielded a unique function among citrullinating enzymes: deimination of peptides with a C-terminal arginine. This activity, which complements that of R-type gingipain virulence factors (gingipain-null mutants are devoid of endogenous citrullination), has been demonstrated for several substrates.

Pathogenic bacteria have evolved sophisticated mechanisms in response to the changing environment and host antimicrobial defense systems. Post-translational modifications are hailed as one of the main factors of pathogens to breach immune tolerance. Among these modifications, citrullination of endogenous proteins seems to be a key process in the initiation of autoimmune reactions. To date, *P. gingivalis* is the only prokaryote that is able to citrullinate proteins and peptides. It has been proposed as a mechanistic link between PD and RA through its potential capacity of generating citrullinated epitopes distinct from endogenous PADs, thus contributing to aggravation of RA. This activity is induced by the sole bacterial peptidylarginine deiminase reported to date, PPAD, which also has a role in the interaction with host cells, so it may be considered as a double target for PD and RA. In contrast, other abundant odontopathogens responsible for PD such as *Prevotella intermedia* and *Fusobacterium nucleatum*, which both lack a PPAD ortholog, do not evince a link with RA.

## Methods

### Protein production, purification, and characterization

*P. gingivalis* PPAD (UniProt database [UP] access code Q9RQJ2 or GenBank entry WP_005873463.1 for NCBI gene tag PG_1424) was obtained through small-scale homologous overexpression as a secreted protein from plasmid-transformed *P. gingivalis* W83 PPAD-deletion mutant strain *Δppad*. Briefly, plasmid pT-COW, which confers resistance against tetracycline^36^, was used as expression vector, and plasmid derivatives encoding the wild type (wt) and a total of 18 PPAD point mutants (W^127^A, D^130^A, D^130^N, R^152^A, R^154^A, R^154^E, T^180^A, G^182^A, H^236^A, H^236^N, D^238^A, D^238^N, C^239^A, C^239^E, C^239^S, N^297^A, C^351^S, and C^351^A; see Table 1) were generated. For this, the wt gene sequence *plus* 1081 upstream base pairs and 267 downstream base pairs was amplified from *P. gingivalis* W83 genomic DNA with primers pTCowPPADf and pTCowPPADr (see Table 1), which contained recognition sequences for restriction endonucleases *Nhe*I and *Sph*I, respectively. The PCR fragment obtained was ligated into pT-COW, previously digested with *Nhe*I and *Sph*I, to yield plasmid pTPP. Point mutations were thereafter introduced into pTPP by the SLIM method^37^ using primers listed under Table 1 and confirmed by DNA sequencing. Plasmid pTPP or its PPAD-mutating variants were introduced into *P. gingivalis* W83 *Δppad* by conjugation, and bacteria were grown under anaerobic conditions (85% N_2_, 5% H_2_ and 10% CO_2_) in liquid Schaedler broth supplemented with hemin (5 mg/ml), menadione (0.5 mg/ml), L-cysteine (50 mg/ml), 1 μg/ml tetracycline, and in the presence or absence of 4,4’-dithiodipyridine (DTDP). Expression levels were monitored by Western-blot analysis. For this, 30 μl of *P. gingivalis* liquid cultures at OD_600_ = 1.0 were separated on SDS-PAGE and transferred to PVDF membranes. Primary PPAD antibodies (kindly provided by Patrick Venables, Oxford) were used in 1:1,000 dilution, secondary HRP-conjugated goat anti-rabbit (Amersham) antibodies were used at 1:10,000 dilution. Cell cultures obtained in the absence of DTDP were used for functional tests (see below). In addition, preparations at a somewhat larger scale—limited by the intrinsic difficulties of cultivating *P. gingivalis*—for structural studies were performed for wt PPAD (DTDP–treated and –untreated) and DTDP-untreated PPAD mutant C^351^A (PPAD–C^351^A) and purified according to^20^.

Protein identity and purity were assessed by 15% Tricine-SDS-PAGE stained with Coomassie blue, peptide-mass fingerprinting of tryptic protein digests (PMF), N-terminal sequencing through Edman degradation, and mass spectrometry (MS). Ultrafiltration steps were performed with Vivaspin 15 and Vivaspin 500 filter devices of 10 kDa cut-off (Sartorius Stedim Biotech). Protein concentrations were estimated applying the respective theoretical extinction coefficients by measuring A_280_ in a spectrophotometer (NanoDrop). Concentrations were also measured by the BCA Protein Assay Kit (Thermo Scientific) with bovine serum albumin as a standard.

### Activity assays

PPAD endo- and exodeimininase activities were determined against kininogen-derived peptides of sequence G-F-S-P-F-R-S-S and P-P-G-F-S-P-F-R, respectively. Briefly, peptides (30 μg) were incubated for 2 h at 37 °C in 100 mM Tris-HCl, pH 7.5 supplemented with 10 mM L-cysteine in the presence of *P. gingivalis* PPAD (0.12, 1.2, 12 and 120 mU) in 30 μl-reaction volumes (final peptide concentration 1 mg/ml). Respective controls were prepared with the same amount of peptide incubated in the reaction buffer alone. Reactions were stopped by addition of 80 μl 0.5% trifluoroacetic acid (TFA) in HPLC-quality water and the samples were further analyzed by HPLC using an ÄKTA Micro chromatography system (GE Healthcare) coupled with an Aeris Peptide XB-C18 4.6/150 column (Phenomenex). Peptides were resolved in 19 column volumes using a 2–80% gradient of phase A (0.1% TFA) and phase B (80% acetonitrile, 0.08% TFA) at 1.5 ml/min flow rate. Eluted peaks were fractionated and citrullination was assessed by MS using a HCT Ultra ETD II ESI Iontrap mass spectrometer (Bruker). To determine the velocity of deimination, peptides were incubated with 0.12 mU (1 h) and 120 mU (2 h) PPAD, respectively, in triplicates. Peak integration data were used to determine the amount of modified peptide in each peak (~11% and ~4.5% for P-P-G-F-S-P-F-Cit and G-F-S-P-F-Cit-S-S, respectively) and estimate the reaction velocity (in pmol·mU^−1^·h^−1^±SD). We found that when P-P-G-F-S-P-F-R became completely citrullinated after overnight incubation (0.12–12 mU), G-F-S-P-F-R-S-S was not modified. Only at ten-fold higher PPAD concentration (120 mU) was certain time-dependent citrillunation of the endosubstrate observed, with 5% of peptide being modified after 2 h. Comparatively, 11% of the exosubstrate was citrullinated after 1 h at a thousand-fold lower PPAD concentration (0.12 mU; see Fig. 3a,b).

Competence of wt and mutant PPADs was assessed by the amount of citrulline produced according to a sensitive colorimetric assay^38^. Results obtained from tree independent assays were adjusted to OD_600_ = 1.0 and presented as % of the activity of pTPP-transformed *Δppad* producing wt PPAD.

### Crystallization and diffraction data collection

Prior to crystallization, DTDP–treated and –untreated wt PPAD and DTDP–untreated PPAD–C^351^A were dialyzed overnight against buffer A (20 mM Tris-HCl, 20 mM sodium chloride, pH 7.5) and further purified by ionic-exchange chromatography on a TSKgel DEAE-2SW column (TOSOH Bioscience) equilibrated with buffer A. A gradient of 4–60% buffer B (20 mM Tris-HCl, 500 mM sodium chloride, pH 7.5) was applied over 30 ml and samples were collected and pooled. Finally, each pool was concentrated by ultrafiltration and subjected to size-exclusion chromatography on a Superdex 75, 10/300 column (GE Healthcare Life Sciences) equilibrated with buffer C (20 mM Tris-HCl, 150 mM sodium chloride, pH 7.5).

Crystallization assays were performed by the sitting-drop vapor diffusion method. Reservoir solutions were prepared by a Tecan robot and 100 nL crystallization drops were dispensed on 96 × 2-well MRC plates (Innovadyne) by a Phoenix nanodrop robot (Art Robbins) or a Cartesian Microsys 4000 XL (Genomic Solutions) robot at the joint IBMB/IRB Automated Crystallography Platform at Barcelona Science Park. Plates were stored in Bruker steady-temperature crystal farms at 4 °C and 20 °C. Successful conditions were scaled up to the microliter range in 24-well Cryschem crystallization dishes (Hampton Research).

The best crystals of wt PPAD with 4-thiopyridine but without substrate (PPAD–TP; *substrate free*) resulting from DTDP treatment during production (see above) were obtained at 20 °C from 1 μl:1 μl drops with protein solution at 20–25 mg/ml concentration in 20 mM Tris-HCl pH 7.4, 100 mM sodium chloride and 100 mM sodium acetate (pH 4.5), 25% [w/v] polyethylene glycol 3,350 as reservoir solution. PPAD mutant C^351^A in complex with the dipeptide methionine-arginine (PPAD–C^351^A+M-R; *substrate complex*) was crystallized similarly but with 100 mM tri-sodium citrate, 20% [w/v] polyethylene glycol 3,000, pH 5.5–6.5 as reservoir solution instead. Finally, wt DTDP-untreated PPAD in complex with the dipeptide aspartate-glutamine (PPAD+D-Q; *substrate-mimic complex*) was crystallized with 100 mM tri-sodium citrate, 2 M ammonium sulfate, pH 5.5–6.5 as reservoir solution. All crystals contained protein spanning A^44^-A^475^ as determined by Edman degradation and MS analysis. Crystals were cryo-protected by rapid passage through drops containing increasing concentrations of glycerol (up to 15% [v/v]). Complete diffraction datasets were collected at 100 K from liquid-N_2_ flash cryo-cooled crystals (Oxford Cryosystems 700 series cryostream) on a Pilatus 6 M pixel detector (from Dectris) at beam line XALOC of ALBA synchrotron (Barcelona, Spain^39^). Further data were collected on the same detector type at beam line ID23-1 of ESRF synchrotron (Grenoble, France) within the Block Allocation Group “BAG Barcelona.” Diffraction data were integrated, scaled, merged, and reduced with program XDS^40^. PPAD–TP, PPAD–C^351^A+M-R, and PPAD+D-Q crystals all contained one protein molecule per asymmetric unit (solvent content, respectively, 41%, 44% and 48%), had the symmetry of the space groups P2_1_2_1_2_1_, C2, and P2_1_2_1_2_1_, respectively, and had different cell constants (see Table 2 for data processing statistics).

### Structure solution and refinement

A similarity search with programs PSI-BLAST and HHPRED identified only low homology models (PDB 3HVM, 1ZBR, 1XKN, 2JER, 3H7C, and 2EWO), which failed to render a solution by conventional molecular replacement and Patterson-search methods. At this point, wt PPAD–TP crystal diffraction data were used for structure solution with ARCIMBOLDO^41^,^42^,^43^. Therefore, 16 datasets with resolutions ranging from 3.0 Å to 1.5 Å from different native protein crystals or heavy-ion soaks with similar cell dimensions were merged with program XPREP. A collection of structure fragments was generated from the six aforementioned distant structural relatives, and ARCIMBOLDO runs were set up in parallel with these fragments and libraries^41^,^42^. These calculations eventually enabled structure solution (see^44^,^45^ for details), and the resulting phase set was subjected to density modification and autotracing with SHELXE^46^, which yielded an improved set of phases and a partial model. These phases and the resulting Fourier map enabled subsequent manual model building with the COOT program^47^, which alternated with crystallographic refinement with PHENIX^48^ and BUSTER/TNT^49^ under inclusion of TLS refinement, until the final refined model of PPAD–TP was obtained. This consisted of residues A^44^-N^464^, one structural sodium ion, seven glycerols, 460 solvent molecules, and 4-thiopyridine moieties respectively attached to the Sγ atoms of C^351^, C^462^, and C^239^. The final Fourier map indicated that the side chain of the latter residue was present in two alternate conformations, one bound to thiopyridine and the other with the sulfur as sulfoxide. See Table 2 for final refinement and model quality statistics.

The structure of PPAD–C^351^A+M-R was solved with PHASER within the PHENIX^50^ package using the refined coordinates of PPAD–TP. The adequately rotated and translated molecule yielded accurate phases, which enabled calculation of an initial Fourier map. Subsequent model completion and refinement proceeded as above. The final model of PPAD–C^351^A+M-R contained residues A^44^-M^463^, one structural sodium cation, a dipeptide of tentative sequence methionine-arginine, five glycerols, one chloride, two azides, 426 solvent molecules, and a free cysteine disulfide-bonded to C^462^. See Table 2 for final refinement and model quality statistics.

The structure of PPAD+D-Q was solved similarly. Model completion and refinement proceeded as above. The final model comprised residues A^44^-E^465^, one sodium cation, a dipeptide of tentative sequence aspartate-glutamine (the distinction between aspartate/asparagine and glutamate/glutamine was performed based on surrounding interacting partners), three glycerols, five phosphates, one chloride, one azide, and 689 solvent molecules. See Table 2 for final refinement and model quality statistics.

### Miscellaneous

Ideal coordinates and parameters for crystallographic refinement of non-standard ligands were obtained from the PRODRG server^51^. Structural similarity searches were performed with DALI^33^, and structure figures were prepared with programs COOT and CHIMERA^52^. Experimental structures were validated with MOLPROBITY^53^. The final coordinates of *P. gingivalis* PPAD–TP (substrate free), PPAD–C^351^A+M-R (substrate complex), and PPAD+D-Q (substrate-mimic complex) are deposited with the PDB at www.pdb.org (access codes 4YT9, 4YTG, and 4YTB).

## Additional Information

**How to cite this article**: Goulas, T. *et al.* Structure and mechanism of a bacterial host-protein citrullinating virulence factor, *Porphyromonas gingivalis* peptidylarginine deiminase. *Sci. Rep.* **5**, 11969; doi: 10.1038/srep11969 (2015).

## Figures and Tables

**Table 1:** Primers used for PPAD single-point mutagenesis.

Name	Sequence (5′−>3′)	Name	Sequence (5′−>3′)
pTCowPPADf	tgcagctagctccttaaggtggatggatatacg	H236Fs	ggcaagtatttggcaccgaa
pTCowPPADr	ctgacgcatgccaatcggtcgttagagttctcc	H236Rs	gatatattcgccgttcggatct
W127AFt	tcttacgctacacgcgactataccggttggttcgcaa	H236AFt	aacgctgtggactgttggggcaagtatttggcaccgaa
W127ARt	gtcgcgtgtagcgtaagagtcagttttcgcaatgatgaa	H236ARt	ccaacagtccacagcgttgatatattcgccgttcggatct
D130Fs	tataccggttggttcgcaa	H236NFt	aacaatgtggactgttggggcaagtatttggcaccgaa
D130Rs	gtcagttttcgcaatgatgaa	H236NRt	ccaacagtccacattgttgatatattcgccgttcggatct
D130AFt	tcttactggacacgcgcttataccggttggttcgcaa	D238AFt	aaccatgtggcttgttggggcaagtatttggcaccgaa
D130ARt	agcgcgtgtccagtaagagtcagttttcgcaatgatgaa	D238ARt	ccaacaagccacatggttgatatattcgccgttcggatct
D130NFt	tcttactggacacgcaactataccggttggttcgcaa	D238NFt	aaccatgtgaattgttggggcaagtatttggcaccgaa
D130NRt	gttgcgtgtccagtaagagtcagttttcgcaatgatgaa	D238NRt	ccaacaattcacatggttgatatattcgccgttcggatct
R152AFs	cctcgtcctaacgatgatga	D239AFt	aaccatgtggacgcttggggcaagtatttggcaccgaa
R152ARs	cacgagacctactttgttcgtatc	D239ARt	ccaagcgtccacatggttgatatattcgccgttcggatct
R152AFt	gactttatttataacgcccctcgtcctaacgatgatga	D239Eft	aaccatgtggacgaatggggcaagtatttggcaccgaa
R152ARt	ggcgttataaataaagtccacgagacctactttgttcgtatc	D239ERt	ccattcgtccacatggttgatatattcgccgttcggatct
R154Fs	gaattccccaaatacgaagc	D239SFt	aaccatgtggactcttggggcaagtatttggcaccgaa
R154Rs	gcggttataaataaagtccacg	D239SRt	ccaagagtccacatggttgatatattcgccgttcggatct
R154AFt	cctgctcctaacgatgatgaattccccaaatacgaagc	N297AFs	acaacagggtatttgttcctg
R154ARt	atcatcgttaggagcagggcggttataaataaagtccacg	N297ARs	tgtacggttgttcattggtg
R154EFt	cctgaacctaacgatgatgaattccccaaatacgaagc	N297AFt	cggcttctctgattctgaacaacagggtatttgttcctg
R154ERt	atcatcgttaggttcagggcggttataaataaagtccacg	N297ARt	tcagaatcagagaagccgtgtacggttgttcattggtg
T180Fs	acatgacggacggatatgga	C351Fs	ggtagcggataagggctatctc
T180Rs	tgagcttcatcccgaacatc	C351Rs	agggcatctgttcctaaccaag
T180AFt	agcaggctggtggcaactacatgacggacggatatgga	C351AFt	gcatgctcgtactcacgaggtagcggataagggctatctc
T180ARt	agttgccaccagcctgcttgagcttcatcccgaacatc	C351ARt	tcgtgagtacgagcatgcagggcatctgttcctaaccaag
G182Aft	agcagaccggtgctaactacatgacggacggatatgga	C351SFt	gcattctcgtactcacgaggtagcggataagggctatctc
G182ARt	agttagcaccggtctgcttgagcttcatcccgaacatc	C351SRt	tcgtgagtacgagaatgcagggcatctgttcctaaccaag

Restriction enzyme recognition sequences are underlined.

**Table 2:** Crystallographic data.

Dataset	PPAD–TP (*substrate free*)	PPAD(C^351^A)+M-R (*substrate complex*)	PPAD+D-Q (*substrate-mimic complex*)
Space group	P2_1_2_1_2_1_	C2	P2_1_2_1_2_1_
Cell constants (a, b, c, in Å; β in °)	58.56, 60.30, 113.68, 90.00	105.36, 59.32, 84.61, 126.60	60.53, 71.31, 105.66, 90.00
Wavelength (Å)	0.9786	0.9795	0.9795
No. of measurements/unique reflections	288,438/64,233	254,674/38,882	1,133,182/90,283
Resolution range (Å) (outermost shell)^a^	42.0–1.50 (1.59 − 1.50)	67.9 − 1.80 (1.90 − 1.80)	46.1 − 1.40 (1.48 − 1.40)
Completeness (%)	98.5 (95.7)	99.3 (98.9)	99.6 (97.5)
R_merge_^b^	0.041 (0.190)	0.062 (0.529)	0.042 (0.305)
R_r.i.m._ [=R_meas_]^c^/ CC(^1^/_2_)^c^	0.047 (0.217)/0.998 (0.973)	0.067 (0.584)/0.999 (0.927)	0.043 (0.321)/1.000 (0.973)
Average intensity^d^	21.0 (7.4)	22.5 (3.7)	36.3 (8.5)
B-Factor (Wilson) (Å^2^)/Aver. multiplicity	25.0/4.5 (4.1)	29.2/6.5 (5.6)	19.3/12.6 (10.1)
Resolution range used for refinement (Å)	42.0 − 1.50	48.6 − 1.80	46.2 − 1.40
No. of reflections used (test set)	64,233 (772)	38,882 (699)	90,283 (903)
Crystallographic R_factor_ (free R_factor_)^b^	0.157 (0.177)	0.156 (0.182)	0.146 (0.149)
No. of protein atoms/solvent molecules/neutral (covalent) ligands/ionic ligands	3,321/460/7 glycerol, 3 *p*-thiopyridine, 1 *S*-oxo/1 Na^+^	3,296/426/5 glycerol, 1 Cys, 1 Met-Arg dipept./1 Na^+^, 2 N_3_^−^, 1 Cl^−^	3,300/689/3 glycerol, 1 imidazole, 1 Asp-Gln dipept./1 Na^+^, 1 N_3_^−^, 1 Cl^−^, 5 PO_4_^3−^
*Rmsd* from target values^e^			
bonds (Å)/angles (°)	0.010/1.03	0.010/0.99	0.010/1.05
Average B-factors (Å^2^)	24.4	28.6	17.4
All-atom contacts and geometry analysis^f^			
Residues			
in favored regions/outliers/all residues	409 (97.6%)/0/419	401 (97.1%)/0/413	411 (97.9%)/0/420
with poor rotamers/bad bonds/bad angles	2 (0.56%)/0/0	3 (0.85%)/0/1 (0.24%)	2 (0.56%)/0/0
with Cβ deviations >0.25 Å/ clashscore	0/3.62 (96^th^ percentile)	1/3.34 (98^th^ percentile)	0/3.47 (96^th^ percentile)
MolProbity score	1.23 (97^th^ percentile)	1.29 (98^th^ percentile)	1.17 (97^th^ percentile)

^a^Data processing values in parenthesis refer to the outermost resolution shell.

^b^For definitions, see Table 1 in^55^.

^c^For definitions, see^56^,^57^.

^d^According to the XDS program^40^. Average intensity is <I/σ(I)> of unique reflections after merging.

^e^According to Engh and Huber^58^.

^f^According to MOLPROBITY^53^,^59^.
